# Successful percutaneous coronary intervention with GuideLiner® catheter for subtotal occlusive lesion in the right coronary artery with anomalous origin from the left sinus of Valsalva: a case report 

**DOI:** 10.1186/s13256-015-0646-0

**Published:** 2015-07-28

**Authors:** Ayumi Shirota, Tetsuya Nomura, Hiroshi Kubota, Shunta Taminishi, Ryota Urata, Takeshi Sugimoto, Yusuke Higuchi, Taku Kato, Natsuya Keira, Tetsuya Tatsumi

**Affiliations:** Department of Cardiovascular Medicine, Nantan General Hospital, 25, Yagi-Ueno, Yagi-cho, Nantan City, Japan

**Keywords:** Anomaly of coronary artery, Complex coronary intervention, GuideLiner, Left sinus of Valsalva

## Abstract

**Introduction:**

Because of the unusual anatomy of an anomalous origin of the right coronary artery from the left sinus of Valsalva, selective cannulation of the guiding catheter in percutaneous coronary intervention for these cases is always challenging.

**Case presentation:**

A 58-year-old Japanese man was admitted to our hospital complaining of worsening exertional chest pain. He was suspected of having unstable angina pectoris and underwent cardiac catheterization. We found a subtotal occlusive lesion in the mid-portion of his right coronary artery that originated from the left sinus of Valsalva. On the previous percutaneous coronary intervention, we failed to cannulate the guiding catheter to the anomalous orifice of the right coronary artery. Therefore, we decided to use the GuideLiner catheter for stable back-up support from the beginning. A 6Fr GuideLiner catheter was introduced into the right coronary artery by anchoring it coaxially with a semi-compliant balloon catheter. And we successfully deployed two drug-eluting stents by crossing over the posterior-descending artery. Final angiography demonstrated favorable dilatation of the target lesion, and native blood flow in the right coronary artery was completely recovered.

**Conclusion:**

GuideLiner is a monorail-type “child” support catheter that facilitates coaxial guiding catheter engagement and an appropriate back-up force, achieving successful device delivery to target lesions in this kind of complex percutaneous coronary intervention.

## Introduction

Anomalies of the coronary artery are a diverse group of congenital disorders that have been investigated by many authors [1, 2]. An anomalous origin of the right coronary artery (RCA) from the left sinus of Valsalva is uncommon but occasionally noted in association with potentially serious sequelae such as sudden death. It has been reported that this type of anomaly is observed in approximately 10% of the population with an anomalous coronary artery [3]. Because of the unusual anatomy, such as the anomalous position of the RCA ostium within the left aortic sinus, a slit-like orifice or atypical angulation, seen in this type of anomalous RCA, selective cannulation of the guiding catheter in percutaneous coronary intervention (PCI) for these cases is always challenging. The GuideLiner catheter (Vascular Solutions Inc., MN, USA), a device which became available in Japan in 2014, is effective for completing PCI even with such an anomalous RCA by coaxially enhancing back-up support for the guiding catheter.

## Case presentation

A 58-year-old Japanese man was admitted to our hospital complaining of worsening exertional chest pain. He had developed acute coronary syndrome (ACS) 2 years previously. At that time, emergent cardiac catheterization demonstrated an occlusive lesion in the mid portion of his RCA and severe stenosis of both the high lateral branch and left circumflex (LCX) artery. The culprit lesion of ACS was considered to be the RCA. However, because the RCA originated from the left sinus of Valsalva, RCA cannulation with a guiding catheter was difficult, and RCA reperfusion in the acute phase could not be achieved. Coronary computed tomography angiography (CTA ) demonstrated that the RCA coursed anteriorly between the aorta and pulmonary artery, the so-called ‘interarterial course’, and then in the atrioventricular (AV) groove to continue its normal distribution. He then underwent coronary artery bypass grafting (CABG). The left internal thoracic artery (LITA) was sequentially grafted from the high lateral branch to the LCX posterolateral branch, and the gastroepiploic artery (GEA) was grafted to the right postero-descending (PD) artery. Routine coronary angiography after surgery demonstrated complete graft patency and unexpected spontaneous reperfusion in the culprit lesion of ACS. Blood flow from the GEA graft and native RCA supplied the proximal lesion of the PD artery. He subsequently remained free from chest symptoms for almost 2 years postoperatively.

In the emergency room of our hospital, his blood pressure was 146/88mmHg, and pulse was 64 beats/minute and regular. A 12-lead electrocardiogram demonstrated small abnormal Q-waves in II, III, and aVF limb leads and no marked ST-T change. Echocardiography showed a slight wall motion abnormality localized in the left ventricular inferior wall with preserved ejection fraction (60.5%). A laboratory study showed no significant abnormal findings. He was suspected of having unstable angina pectoris and underwent cardiac catheterization.

**Fig. 1:**
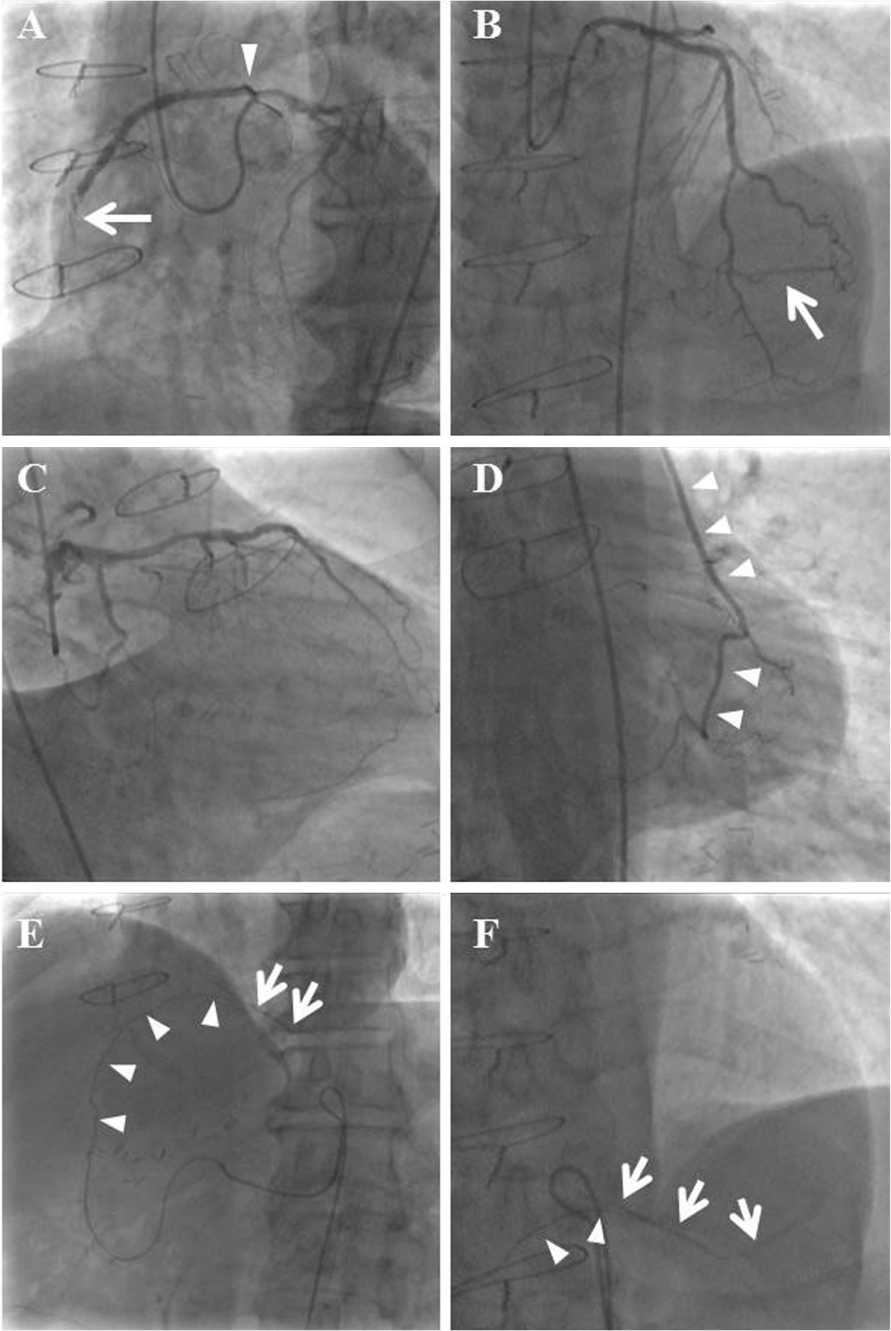
The Amplatz left 2 catheter could be engaged in both the right (**a**) and left (**b**, **c**) coronary artery. **a** The right coronary artery originated from the left sinus of Valsalva (*arrowhead*). The mid right coronary artery was subtotally occluded (*arrow*). **b** The left anterior descending artery was intact. A clear collateral artery from the second diagonal branch to the distal right atrioventricular branch was observed (*arrow*). **c** A caudal image of the left coronary artery shows severe stenosis of the high lateral branch and left circumflex artery. **d** The left internal thoracic artery graft (*arrowheads*) to left circumflex artery was patent. **e**, **f** The gastroepiploic artery graft (*arrowheads*) to right postero-descending artery (*arrows*) was patent. **f** is a magnified image of **(e)**

A 5Fr Amplatz left 2 catheter (Goodman Co. Ltd., Aichi, Japan) could be engaged in both the right (Fig. 1a) and left (Fig. 1b, c) coronary arteries. Blood flow both in the left anterior descending artery (LAD ; Fig. 1b, c) and from the LITA to LCX artery (Fig. 1d, arrowheads) was favorable. Grade 2 collateral channels from the second diagonal branch to the distal right AV branch were also visualized (Fig. 1b, arrow). The RCA originated from the left coronary cusp just below the ostium of the left coronary artery (LCA; Fig. 1a, arrowhead). A subtotal occlusive lesion in the mid portion of the RCA was observed (Fig. 1a, arrow). The graft from the GEA (Fig. 1e, f, arrowheads) to right PD artery (Fig. 1e, f, arrows) was patent, but it had markedly reduced in size. Therefore, blood supply for the area perfused with the right PD artery was very limited. Because collateral blood flow to the distal right AV branch was also insufficient, the area distal to the subtotal occlusive lesion in the mid RCA was considered to be at risk. However, because the patient had never felt chest symptoms before his ACS episode 2 years previously, the anomalous interarterial course of the RCA in this patient was not thought to have been involved in the etiology of myocardial ischemia. As stated above, the atherosclerotic subtotal occlusive lesion in the mid RCA was eventually considered to be the cause of his chest symptom this time, and we decided to perform PCI for this lesion.

**Fig. 2:**
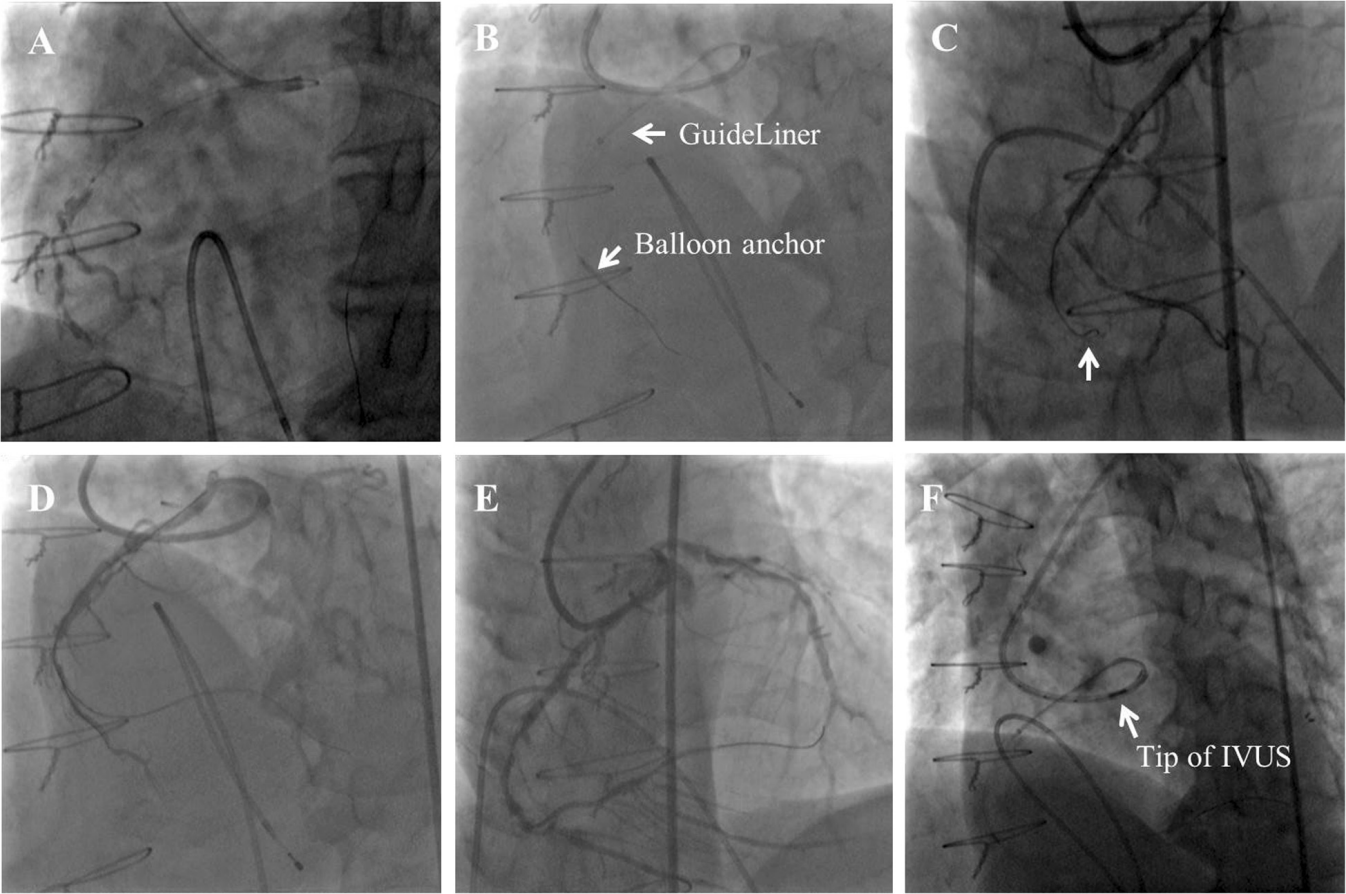
**a** Selective right coronary artery angiography by tip injection from the Mizuki microcatheter. **b** The GuideLiner catheter was introduced into the right coronary artery with a coaxial balloon anchoring it in the right ventricular branch. **c** Sion guidewire (*arrow*) could not pass through the subtotal occlusive lesion. **d** Fielder XT-A finally crossed the lesion. **e** The right coronary artery after pre-dilation with φ2 -mm balloon from the mid right coronary artery through to the proximal atrioventricular branch. **f** The Eagle Eye Platinum intravascular ultrasonography catheter could not be advanced beyond the first curve of the Judkins left 4 guiding catheter inside the GuideLiner. *IVUS* intravascular ultrasonography

On the previous PCI for ACS, we failed to cannulate the guiding catheter to the anomalous RCA orifice. Therefore, we decided to use the GuideLiner catheter for stable back-up support from the beginning. We started the PCI procedure with a right transfemoral approach while inserting a temporary pacing catheter from the right femoral vein, because we were concerned that PCI might cause distal embolization and bradycardia if the target lesion consisted of vulnerable plaque or fresh thrombi. First, a 7Fr Sherpa NX Judkins left (JL) 4 guiding catheter (Medtronic Inc., MN, USA) was inserted through the LCA and Sion blue (Asahi Intecc Co. Ltd., Aichi, Japan) was passed through the LAD artery. Then, we slightly disengaged the guiding catheter from the LCA ostium and attempted to direct the catheter tip towards the anomalous RCA ostium by rotating it clockwise. Sion (Asahi Intecc Co. Ltd., Aichi, Japan) with the Mizuki microcatheter (Kaneka Corp., Osaka, Japan) could then pass into the anomalous RCA and was deposited in the right ventricular (RV) branch (Fig. 2a). Then, a 6Fr GuideLiner catheter was introduced into the RCA by anchoring it coaxially with a 2.0-mm semi-compliant balloon catheter in the RV branch (Fig. 2b). We could not pass Sion with the Mizuki microcatheter through the subtotal occlusive lesion (Fig. 2c, arrow), so we changed the guidewire to Fielder XT-A (Asahi Intecc Co., Ltd., Aichi, Japan), and then finally passed the lesion (Fig. 2d).

**Fig. 3:**
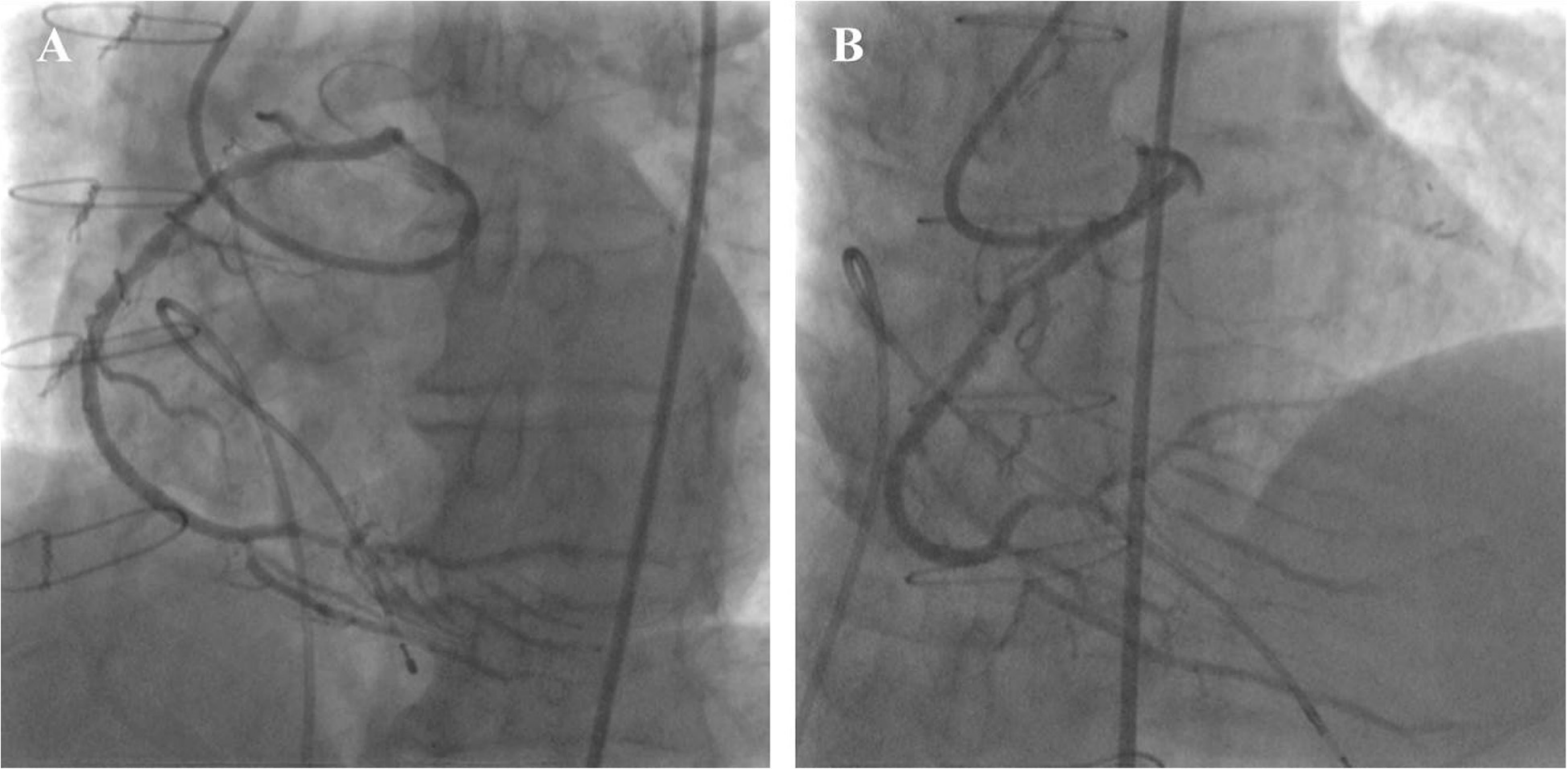
Final angiography demonstrated favorable dilatation from the mid right coronary artery through to proximal atrioventricular branch. An image from the left anterior oblique position (**a**). An image from the anteroposterior cranial position (**b**)

After 2.0-mm semi-compliant balloon inflation from the mid RCA through to the proximal AV branch (Fig. 2e), we attempted to advance an Eagle Eye® Platinum intravascular ultrasonography (IVUS) catheter (Volcano Corp., CA, USA) to the lesion. However, resistance to its insertion was so marked that we could not entirely advance the IVUS catheter over the first curve of the JL4 inside the GuideLiner (Fig. 2f). We tried to change the position of the GuideLiner, but ultimately failed to deliver the IVUS catheter into the RCA. From the angiographical findings, we predicted the vessel size and initially deployed Xience Xpedition® (Abbott Laboratories, IL, USA) 2.5/28mm by crossing over the PD artery. We additionally deployed Xience Xpedition® 3.0/38 mm slightly overlapping the proximal edge of the first drug-eluting stent. Final angiography demonstrated favorable dilatation from the mid RCA through to the proximal AV branch, and native blood flow in the RCA was completely recovered (Fig. 3a, b).

The patient effectively tolerated the entire procedure and we successfully completed the treatment. Total procedure time was 180 minutes. Irradiation time was 110 minutes and accumulated irradiation dose was 73.8mGy-m^2^. Consumed volume of contrast medium was 287.3mL. Pre-procedural estimated glomerular filtration rate was 64.7 mL/minute/1.73m^2^, and worsening renal function was not observed after the procedure. His chest symptom fully resolved after this intervention. He followed an uneventful hospital course and was consequently discharged; no adverse events requiring hospitalization have been observed for almost 1 year since this intervention.

## Discussion

We encountered a rare case of a subtotal occlusive lesion in the mid-portion of the RCA that had originated from the left sinus of Valsalva. This type of anomaly is a rare but potentially life-threatening abnormality of the coronary circulation, even in the absence of coronary atherosclerosis [4]. An aberrant RCA interarterial course often seen in this anomaly can cause ostial occlusion due to aortic expansion during exercise, and may result in myocardial ischemia. However, because the patient had never felt chest symptoms before his ACS episode, we did not consider that myocardial ischemia in this patient was due to the aberrant RCA interarterial course. There are three treatment options for symptomatic patients with anomalous coronary arteries: medication [5], PCI [6], and surgical treatment [7]. Because the cause of ischemia in this case was strongly suspected to be the organic subtotal occlusive lesion in the mid RCA and the patient had a history of bypass graft failure, we decided to perform PCI and not redo a CABG in this case.

There have been several reports on PCI for such an anomalous RCA; however, PCI for those cases is always accompanied by difficulty in achieving coaxial alignment with the anomalous RCA orifice. Although several guiding catheters for PCI with size and tip-shape variations are now available, it is usually difficult to achieve stable catheterization in a coronary artery with an anomalous origin. Unstable guiding catheter insertion to the coronary artery leads to reduced back-up support, tending to cause PCI failure. Appropriate guiding catheter selection is thought to be critical to ensure successful PCI in such cases [8]. There have been some reports discussing an anomalous origin of the RCA within the aortic root and how this anatomy may influence decision making regarding selection of a guiding catheter for PCI [9, 10].

In general, during complex PCI procedures, we sometimes encounter a situation whereby device delivery to the lesion is difficult because of the weaker back-up force with the guiding catheter. The prevailing management methods in such cases are as follows: (1) select a guiding catheter offering greater back-up support (sizing up or change the tip shape), (2) balloon anchor technique (side branch or coaxial anchoring), (3) change to a support-type guidewire, and (4) child-in-mother technique. It is widely recognized that the child-in-mother technique is effective for complex PCI. The child catheter inside the mother guiding catheter is introduced deeply into the coronary artery, which promotes the back-up force of the guiding catheter and devices can be smoothly delivered to the target lesion. However, this system is usually complicated because of its over-the-wire system, and there is sometimes concern that the length of device catheters such as a balloon or stent is too short because they are usually used through a long shaft child catheter. There is further concern over thrombus formation between the mother and child catheters.

The GuideLiner catheter, released in Japan in 2014, is a monorail-type “child” support catheter that comprises a 25-cm silicon-coated guide extension catheter connected via a metal “collar” with a 125-cm stainless steel shaft to a proximal positioning tab. It can be advanced over the guidewire through the hemostatic valve without the need to disconnect the valve from the mother guiding catheter. The catheter tip is then advanced beyond the tip of the mother guide into the coronary artery by pushing on the proximal tab, which has been approved to provide extra support and coaxial guiding catheter engagement, and can facilitate device delivery to target lesions. This device has made up for the shortcomings of the conventional child-in-mother technique described above by adopting the monorail system. Since GuideLiner catheter use was first reported in humans in 2010 [11], several reports have demonstrated that it can be utilized effectively for complex PCI [12, 13].

PCI for the anomalous RCA in this patient was the second attempt. We did not succeed in selecting an optimal guiding catheter that could aid with completing the PCI procedure on the first try at the time of ACS 2 years ago. On this second attempt, we thought that it would be a waste of time, energy, and guiding catheters to single out an optimal guiding catheter. Therefore, from the beginning, we decided to use the epoch-making device “GuideLiner” to cannulate the anomalous RCA orifice selectively. There has been only one report describing the usefulness of this GuideLiner catheter for PCI for a stenotic lesion in an anomalous RCA [14]. However, the target lesion in our case was a subtotal occlusive lesion and much stronger back-up support was needed for successful PCI compared with PCI for the stenotic lesion in the previous report. Actually, we found it to be very effective for completing the PCI procedure to coaxially enhance back-up support for the guiding catheter.

However, demerits of the GuideLiner catheter compared to the conventional child-in-mother technique have been noted. Operators may feel resistance when devices pass through the rapid exchange collar portion of the GuideLiner inside the guiding catheter. Therefore, there is a concern that stent fracture may occur when the folding stent is stuck in the collar portion of the GuideLiner [15]. Also, the inner lumen size of 6Fr GuideLiner is 0.056 inches (1mm ), which is smaller than that of the 5Fr ordinary child catheter. For example, both the 5Fr DIO guiding catheter (Goodman Co. Ltd., Aichi, Japan) and 5Fr Heartrail ST01 guiding catheter (Terumo Corp., Tokyo, Japan) have a 0.059-inch (1.5mm ) inner lumen size. Therefore, device passage inside the GuideLiner catheter can be hampered. Although we could fortunately deliver stents to the target lesions with no resistance at the collar portion and inside the GuideLiner in this procedure, the Eagle Eye® Platinum IVUS catheter could not completely cross the first curve of the JL guiding catheter inside the GuideLiner. To overcome this, we should have used other more slender or flexible imaging modalities. However, during all of the procedures except for delivering the IVUS catheter, GuideLiner was very important in providing stable back-up support for the guiding catheter.

## Conclusions

Here, we encountered a complex PCI case with a subtotal occlusive lesion in the mid RCA that originated from the left sinus of Valsalva. The GuideLiner catheter facilitates coaxial guiding catheter engagement and an appropriate back-up force, which can facilitate device delivery to target lesions in this kind of complex coronary intervention.

## Consent

Written informed consent was obtained from the patient for publication of this case report and accompanying images. A copy of the written consent is available for review by the Editor-in-Chief of this journal.

## Abbreviations

ACS: Acute coronary syndrome
AV: Atrioventricular
CABG: Coronary artery bypass grafting
GEA: Gastroepiploic artery
IVUS: Intravascular ultrasonography
JL: Judkins left
LAD: Left anterior descending artery
LCA: Left coronary artery
LCX: Left circumflex
LITA: Left internal thoracic artery
PCI: Percutaneous coronary intervention
PD: Postero-descending
RCA: Right coronary artery
RV: Right ventricular
